# Determination of Ram (*Ovis aries*) Sperm DNA Damage Due to Oxidative Stress: 8-OHdG Immunodetection Assay vs. SCSA^®^

**DOI:** 10.3390/ani12233286

**Published:** 2022-11-25

**Authors:** Pedro Javier Soria-Meneses, Alejandro Jurado-Campos, Virgilio Gómez-Rubio, Irene Sánchez-Ajofrín, Ana Josefa Soler, José Julián Garde, María del Rocío Fernández-Santos

**Affiliations:** 1 SaBio IREC (CSIC-UCLM-JCCM), Campus Universitario, s/n, 02071 Albacete, Spain; 2 Departamento de Matemáticas, ETSIIA, UCLM, Campus Universitario, s/n, 02071 Albacete, Spain; 3 Faculty of Pharmacy, UCLM, Dr. José María Sánchez Ibáñez, s/n, 02008 Albacete, Spain

**Keywords:** 8-OHdG, SCSA^®^, ram sperm, DNA, oxidative stress, flow cytometry

## Abstract

**Simple Summary:**

The particular architecture and biology of spermatozoa make them highly susceptible to oxidative stress, which can lead to DNA decondensation and fragmentation. It might also induce the formation of 8-OHdG, which is an early marker of DNA damage caused by oxidative stress. Because ruminant sperm DNA is highly compacted, it is rare to detect damage in ram DNA by conventional techniques. In this study, our aim was to evaluate the efficacy of detecting oxidative DNA damage in ram sperm samples using SCSA^®^ vs. an 8-OHdG immunodetection assay. Our results showed that SCSA^®^ and an oxidative-stress-specific 8-OHdG immunodetection assay can detect DNA damage caused by oxidative stress in ram sperm cells under high oxidative conditions; therefore, it is not necessary to use an oxidative-stress-specific technique to detect DNA damage in ovine spermatozoa.

**Abstract:**

Conventional DNA analysis techniques can hardly detect DNA damage in ruminant spermatozoa due to high DNA compaction in these cells. Furthermore, these techniques cannot discriminate whether the damage is due to oxidative stress. The main purpose of this study was to evaluate the efficacy of two techniques for determining DNA damage in ovine sperm when the source of that damage is oxidative stress. Semen samples from twenty Manchega rams (*Ovis aries*) were collected and cryopreserved. After thawing, the samples were subjected to different levels of oxidative stress, and DNA oxidation was quantified using an 8-hydroxy-2′-deoxyguanosine (8-OHdG) immunodetection assay and Sperm Chromatin Structure Assay (SCSA^®^). For this purpose, we evaluated five different concentrations of an oxidation solution (H_2_O_2_/FeSO_4_•7H_2_O) on ram sperm DNA. Our study with the 8-OHdG immunodetection assay shows that there are higher values for DNA oxidation in samples that were subjected to the highest oxidative stress (8 M H_2_O_2_/800 µM FeSO_4_•7H_2_O) and those that were not exposed to high oxidative stress, but these differences were not significant (*p* ≥ 0.05). The two SCSA^®^ parameters considered, DNA fragmentation index (DFI %) and high DNA stainability (HDS %), showed significant differences between samples that were subjected to high concentrations of the oxidation agent and those that were not (*p* < 0.05). We can conclude that the 8-OHdG immunodetection assay and SCSA^®^ detect DNA damage caused by oxidative stress in ovine sperm under high oxidative conditions; SCSA^®^ is a more straightforward method with more accurate results. For these reasons, an oxidative-stress-specific assay such as 8-OHdG immunodetection is not needed to measure DNA damage caused by oxidative stress in ram sperm samples.

## 1. Introduction

Spermatozoa are exposed to a higher risk of oxidative DNA damage than any other kind of cell because of their particular architecture and biology, and oxidative alterations could lead to DNA fragmentation and nuclear decondensation [1,2]. Nuclear DNA is protected from oxidative damage because, during spermiogenesis, histones are removed and replaced by protamines. These molecules allow a high level of compaction of sperm DNA [3]. Despite the compaction of nuclear DNA, there are areas of the mammalian nuclear genome that are vulnerable to oxidative attacks that correspond to the less dense genomic regions still organized into nucleosomes. These regions are most vulnerable to oxidative DNA damage [4].

Because ruminant sperm DNA is highly compacted, damage to ovine sperm DNA is seldom detectable by conventional methods. The most widely used techniques of measuring DNA fragmentation index (DFI) are Sperm Chromatin Structure Assay (SCSA^®^) [5], the terminal deoxynucleotidyl transferase dUTP nick labelling (TUNEL) assay [6], the sperm chromatin dispersion (SCD) test [7], and the comet assay [8,9].

The comet assay and SCD test were introduced as microscope tests and so do not require flow cytometry [10]. Analytical techniques such as computer-assisted sperm analysis and fluorescence microscopy allow us to evaluate a few hundred spermatozoa, which is not considered representative of the ejaculate [11]. Consequently, a larger number of cells (in the order of thousands of spermatozoa) should be considered for sperm analysis. Therefore, flow cytometry is increasingly used, replacing time-consuming and error-prone analysis techniques, allowing the evaluation of thousands of spermatozoa in a few seconds, resulting in a more representative evaluation of the sample [11,12,13]. The TUNEL assay and SCSA^®^ are two of the most widely used techniques to assess chromatin status, but they have some differences [14]. Whereas the TUNEL assay does not require pre-treatment to measure the single- and double-strand breaks in DNA, SCSA^®^ requires prior denaturation [14,15]. However, the TUNEL assay is more complicated to perform, as it involves washes, DTT treatment, paraformaldehyde fixation, permeabilization with Triton X, and the use of enzymes, which translates into more steps to provide only one parameter [10,14].

Therefore, the most commonly used technique to assess sperm chromatin status by flow cytometry is SCSA^®^ based on the susceptibility of sperm DNA to acid-induced denaturation in situ and on the subsequent staining with the metachromatic fluorescent dye acridine orange (AO), which intercalates easily into the DNA. This assay is a tool for measuring the important properties of sperm nuclear chromatin integrity [16]. AO fluorescence shifts from green, when AO is associated with double-stranded DNA (dsDNA), to red, when associated with single-stranded DNA (ssDNA). ssDNA breakage formation is induced by the denaturation step; thus, each sperm head yields a mixture of green and red fluorescence when interrogated with a 488 nm laser, depending on the susceptibility of chromatin to denaturation and DNA fragmentation (number of nicks). For each spermatozoon, data are processed to obtain two parameters with this technique: DNA fragmentation index (DFI), which is the ratio of red fluorescence vs. the total intensity of the fluorescence (red/[red + green] × 100), representing the shift from green to red fluorescence, and high DNA stainability (HDS), representing sperm with increased green fluorescence. High values of DFI indicate chromatin abnormalities, and high HDS values are characteristic of immature sperm and/or sperm with altered protein composition [5].

SCSA^®^ is a simple test to assess sperm DNA damage, but it does not differentiate whether the DNA damage is caused by oxidative stress or other factors such as changes in the environment, which include environmental heat; the presence of S=S bonds between nuclear spermatic protamines, exposure to growth hormones, and aromatic hydrocarbons; and diseases such as cancer [10,17,18,19]. In order to detect DNA damage explicitly caused by oxidative stress, Vorilhon et al. (2018) conducted a study to detect the presence of a DNA oxidation marker (8-hydroxy-2′-deoxyguanosine) through different immunodetection protocols to establish a discriminating threshold for oxidative DNA damage in human sperm [20]. The 8-OHdG immunodetection assay is based on the detection of an early marker of DNA oxidation, 8-hydroxy-2′-deoxyguanosine (8-OHdG) [21]. Of the four DNA bases, guanine, due to its low oxidation potential [22], is the most susceptible to oxidation, and the interaction with hydroxyl radical (OH•) leads to the formation of C8-hydroxyguanosine (8-OHGua), or its nucleoside, from deoxyguanosine (8-hydroxy-2′-deoxyguanosine). Initially, the reaction of OH• addiction leads to the generation of radical adducts, and then the 8-OHdG is formed by one electron abstraction [18]. Mammalian cells contain a coordinated base excision repair pathway (BER) for the removal of oxidized residues, such as 8-OHdG, via 8-Oxoguanine DNA glycosylase 1 (OGG1), which is a bifunctional N-glycosylase/DNA lyase enzyme. OGG1 activity leads to the formation of an apurinic site (AP site), as well as a nick in the phosphodiester backbone, yielding a 3′α,β-unsaturated aldehyde and a 5′deoxyribosephosphate [17,20]. Apurinic endonuclease 1 (APE1) then cleaves the AP site to form the 3′-OH group adjacent to the 5′deoxyribophosphate in preparation for inserting a new guanine nucleotide by polymerase β. The base excision repair pathway is completed by the phosphodiesterase activity of ligase III, which seals the nick in the backbone. The non-presence of APE1 in sperm cells makes it impossible to complete the BER pathway [17]. The non-resolution of sperm DNA 8-OHdG adducts could persist in the zygote and create the opportunity for mutations to occur prior to the initiation of embryonic development [23,24].

Recently, we have shown that the 8-OHdG immunodetection assay only detected oxidative stress damage in ram sperm samples if the spermatozoa were subjected to a very high oxidative treatment [25].

In the present study, we aim to evaluate the efficacy in detecting oxidative stress damage in ram sperm samples using SCSA^®^ vs. the 8-OHdG immunodetection technique. Our objective is to check whether a specific technique for detecting DNA damage due to oxidative stress is necessary for ruminant samples, which have highly compacted DNA, or whether the SCSA^®^ technique offers similar results.

## 2. Materials and Methods

### 2.1. Reagents and Media

All the chemicals were acquired from Merck (Madrid, Spain) except for monoclonal anti-8-OHdG antibody (mouse anti-8-OHdG monoclonal antibody DNA/RNA damage antibody-15A, NB110-96878, Novus Biologicals^®^, Lille, France), Alexa Fluor™ 488 goat anti-mouse antibody (Fisher Scientific, Madrid, Spain), Hoechst 33342 (ThermoFisher Scientific, Madrid, Spain) and acridine orange (Polysciences, Inc., Warrington, PA, USA). Flow cytometry consumables, equipment, and software were purchased from Beckman Coulter (Fulerton, CA, USA). The freezer extender, Biladyl^®^, was purchased from Minitube (Tiefenbach, Germany).

### 2.2. Animal Ethics and Sperm Collections

Semen samples were collected using an artificial vagina from twenty healthy males of Manchega sheep breed (>3 years of age) housed at the Experimental Farm of the University of Castilla-La Mancha and the Regional Centre for Animal Selection and Reproduction in Valdepeñas (CERSYRA), which are part of the Manchega sheep breed selection scheme. Animal handling was performed in accordance with Spanish Animal Protection Regulation, RD 53/2013, which conforms to European Union Regulation 2010/63. Volume, concentration, wave motion (0 no movement to 5 strong wave motion), and sperm motility were assessed shortly after collection. Only ejaculates with wave motion values of 4 or 5 and sperm motility higher than 80% were frozen. 

Samples were cryopreserved in Biladyl^®^, a commercial freezing extender, with 7% glycerol and 20% egg yolk. Initially, semen was extended to 400 × 10^6^ sperm/mL in fraction A of Biladyl^®^, and slowly cooled from 30 to 5 °C over 2 h in a programmable temperature controller (PolyScience^®^). Then, semen samples were extended to 200 × 10^6^ sperm/mL in fraction B of Biladyl^®^, with 7% of glycerol. After 2 h of equilibration at 5 °C, semen was automatically packed into 0.25 mL straws and frozen in a programmable biofreezer (Planer Kyro 10 Series III; Planer PLC, London, UK) following a freezing curve (−20 °C/min from 5 °C to −100 °C and −10 °C/min from −100 °C to −140 °C). Cryopreserved semen was immersed into liquid nitrogen and stored in a liquid nitrogen container [26]. The samples are part of the germplasm bank of the Manchega sheep breed, and all of them were frozen for at least 1 year.

### 2.3. Experimental Design

Two techniques for measuring DNA damage were compared, 8-OHdG immunodetection assay and SCSA^®^. Therefore, five different concentrations of oxidation solution were used: 1 mM H_2_O_2_/0.1 µM FeSO_4_•7H_2_O (OS1); 10 mM H_2_O_2_/1 µM FeSO_4_•7H_2_O (OS2); 100 mM H_2_O_2_/10 µM FeSO_4_•7H_2_O (OS3); 1 M H_2_O_2_/100 µM FeSO_4_•7H_2_O (OS4); and 8 M H_2_O_2_/800 µM FeSO_4_•7H_2_O (OS5).

Sperm samples were thawed for 30 s at 37 °C and washed in PBS. Each PBS-washed sample was divided into six aliquots and each aliquot was subjected to a different concentration of oxidant solutions (room temperature, 1 h). Then, the 8-OHdG immunodetection assay and SCSA^®^ were carried out.

#### 2.3.1. 8-OHdG Immunodetection Assay

**Figure 1:**
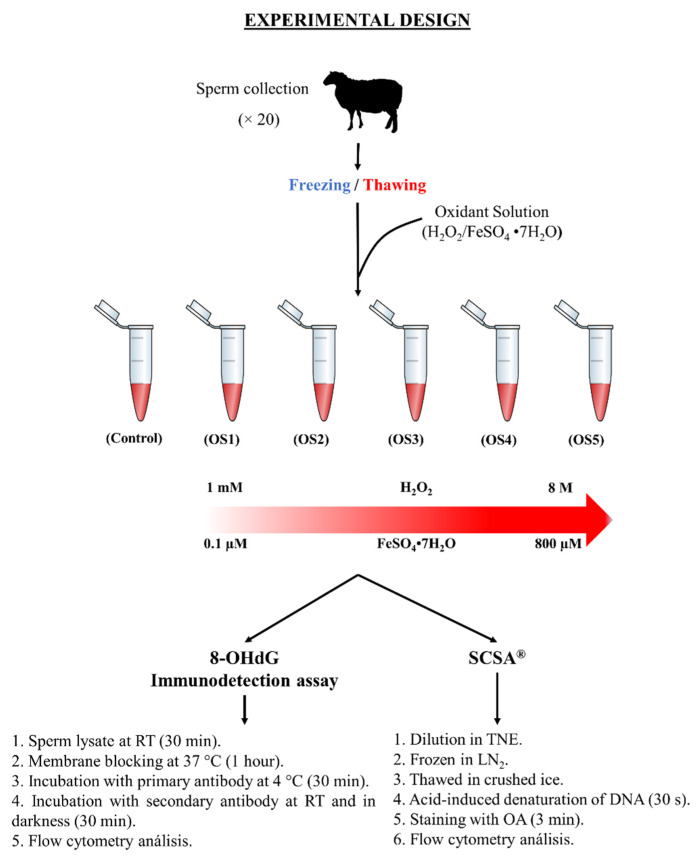
Experimental design of the study to assess the accuracy of ram sperm DNA oxidation quantification using an 8-OHdG immunodetection assay and chromatin stability using Sperm Chromatin Structure Assay (SCSA^®^).

PBS-washed samples were incubated for 30 min in the dark in a lysis buffer comprising PBS, 2 mM dihidrothreitol (DTT), and 0.5% Triton X-100. Then, samples were washed with PBS, and the membranes were blocked in 3% BSA-PBS at 37 °C and incubated in the dark for 1 h. Afterward, samples were divided into different tubes and incubated with 1:1000 monoclonal anti-8-OHdG antibody for 30 min (4 °C, in darkness). Samples were washed and incubated with Alexa Fluor™ 488 goat anti-mouse antibody (30 min, room temperature, and in darkness). Sperm samples were washed twice and diluted in PBS with 3 µM Hoechst 33342 to 1 × 10^6^ spermatozoa/mL. Each sample was incubated for 1 h with the oxidant solution (Figure 1). This protocol for the immunodetection of 8-OHdG was proposed by de Iuliss et al. (2009) and Vorilhon et al. (2018) for human sperm and modified by Soria-Meneses et al. (2019) for ovine sperm [20,21,25].

#### 2.3.2. Sperm Chromatin Structure Assay (SCSA^®^)

Chromatin stability was assessed following SCSA^®^ [5]. PBS-washed samples were diluted in TNE buffer (0.01 M Tris-HCl, 0.15 M NaCl, 1 mM EDTA, and pH 7.4) to a final sperm concentration of 2 × 10^6^ cells/mL, frozen immediately in liquid nitrogen, and stored in a freezer at −80 °C until analysis. For evaluation, the samples were thawed in crushed ice. Acid-induced denaturation of DNA in situ was achieved by adding 0.4 mL of an acid detergent solution (0.15 M NaCl, 0.08 N HCl, 0.17% Triton X-100, and pH 1.4) to 200 μL of the sample. After 30 s, the cells were stained by adding 1.2 mL of an AO solution (0.2 M Na_2_HPO_4_, 0.15 M NaCl, 0.1 M citric acid, 1 mM EDTA, 6 μg/mL AO, and pH 6.0). The stained samples were analysed by flow cytometry exactly 3 min after adding the AO solution. A tube with 0.4 mL of acid detergent solution and 1.2 mL of AO solution was run through the system before any samples were assessed and between sample assessment. At the beginning of each session, a standard semen sample was run through the cytometer, and settings were adjusted so that mean fluorescence values (0–1023 linear scale) for FL-1 (green fluorescence) and FL-3 (red fluorescence) were 475 and 125, respectively. Results of the DNA denaturation test were processed to obtain the DFI, i.e., the ratio of red fluorescence vs. total intensity of the fluorescence (red/[red + green] × 100) for each spermatozoon, representing the shift from green to red fluorescence. High values of DFI indicate chromatin abnormalities. Flow cytometry data were processed to obtain % DFI (% of spermatozoa with DFI > 25) and % HDS, which is the percentage of spermatozoa with green fluorescence higher than channel 600 of 1024 channels [5,27] (Figure 1).

### 2.4. Flow Cytometry Analysis

The 8-OHdG immunodetection assay was carried out on a CytoFlex S (Beckman Coulter, Inc.) equipped with violet (405 nm) and blue (488 nm) lasers for the excitation of Hoechst 33342 and Alexa Fluor 488, respectively. Alexa Fluor 488 has a maximum emission at 520 nm and a FITC photodetector (525/40 band-pass filter) was used, while Hoechst 33342 has a maximum emission at 461 nm and a PB450 photodetector (450/45 band-pass filter) was used. The flow cytometry data were analysed using the software CytoExpert version 2.3.0.84 (Beckman Coulter, Inc.). SCSA^®^ was carried out on a Cytomics FC-500 (Beckman Coulter, Inc.) equipped with a 488 nm argon ion laser for the excitation of AO. AO green fluorescence was detected with a 530/28 band-pass filter (FL-1), while AO red fluorescence was detected with a 620/40 band-pass filter (FL-3). The analysis of the data was carried out using the software WEASEL version 2.4. Non-sperm events, such as bacteria or extender particles, were discarded by gating in an FSC (forward scatter of the laser light)/SSC (side scatter of the laser light) dot plot based on differences in complexity and size among debris and sperm cells [28].

### 2.5. Statistical Analysis

The R statistical package was used to perform the statistical analysis of this study [29]. We used linear mixed-effects models to analyse the effects of different oxidant agent concentrations (fixed factors) on ram sperm DNA, and a random effect on the male was also included in the model. The ‘lme4’ package was used to fit all mixed-effects models [30]. The results are presented as mean ± standard error of the mean (SEM), and statistical significance was considered for *p* < 0.05. The bars in the plots show the approximate 95% confidence intervals computed using mean ± t0.975, n-1*SEM. This is a conservative confidence interval, as it is difficult to estimate the number of degrees of freedom for these mixed-effects models [31]. Furthermore, the R package ‘multcom’ and Tukey’s correction were used to adjust the *p*-values and account for multiple testing when comparing the effects of the different variables [32].

## 3. Results

In the present work, we assessed oxidative stress damage in sperm DNA using two different techniques: the 8-OHdG immunodetection assay and SCSA^®^ carried out by flow cytometry. To conduct this study, five different concentrations of an oxidant solution were tested on sperm samples: 1 mM H_2_O_2_/0.1 µM FeSO_4_•7H_2_O (OS1); 10 mM H_2_O_2_/1 µM FeSO_4_•7H_2_O (OS2); 100 mM H_2_O_2_/10 µM FeSO_4_•7H_2_O (OS3); 1 M H_2_O_2_/100 µM FeSO_4_•7H_2_O (OS4); and 8 M H_2_O_2_/800 µM FeSO_4_•7H_2_O (OS5).

### 3.1. 8-OHdG Immunodetection Assay

**Figure 2:**
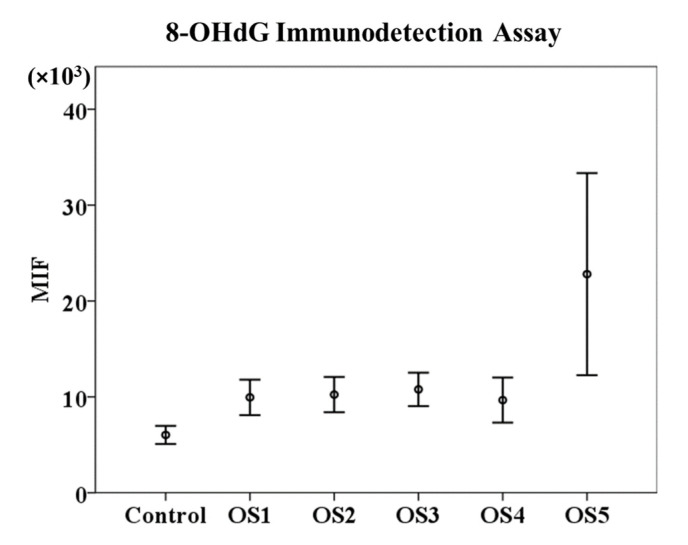
DNA oxidation quantification using an 8-OHdG immunodetection assay. Mean of Alexa Fluor 488 intensity of fluorescence ± standard error of the mean (MIF ± SEM) differences from 8-OHdG immunodetection assay. OS1 = 1 mM H_2_O_2_/0.1 µM FeSO_4_•7H_2_O; OS2 = 10 mM H_2_O_2_/1 µM FeSO_4_•7H_2_O; OS3 = 100 mM H_2_O_2_/10 µM FeSO_4_•7H_2_O; OS4 = 1 M H_2_O_2_/100 µM FeSO_4_•7H_2_O; and OS5 = 8 M H_2_O_2_/800 µM FeSO_4_•7H_2_O.

In the first part of this study, we compared the results of the mean intensity of fluorescence (MIF) of Alexa Fluor 488 obtained from the sample’s incubation with different concentrations of the oxidation solution H_2_O_2_/FeSO_4_•7H_2_O using the 8-OHdG immunodetection assay. Our results show that the OS5 treatment had the highest value of MIF (22,798.94 ± 2246.99) compared to the rest of the treatments (OS1 = 9947.89 ± 403.09; OS2 = 10,233.45 ± 393.18; OS3 = 10,776.14 ± 379.40; and OS4 = 9661.457 ± 513.53) and the control (6032.22 ± 452.99). However, these differences were not significant (*p* ≥ 0.05) (Figure 2).

**Figure 3:**
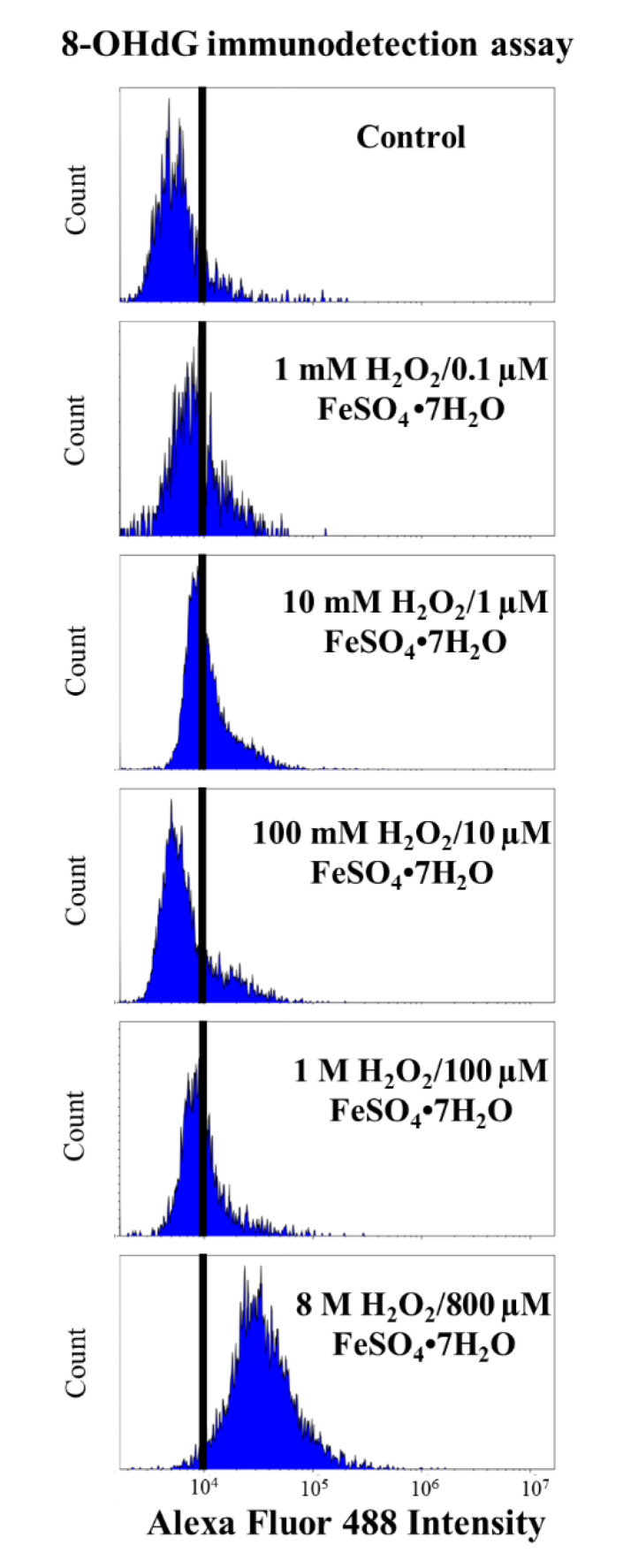
Histograms of DNA oxidation quantification using an 8-OHdG immunodetection assay. Basal fluorescence of evaluated subjects (control) and samples submitted to 1 mM H_2_O_2_/0.1 µM FeSO_4_•7H_2_O (OS1); samples submitted to 10 mM H_2_O_2_/1 µM FeSO_4_•7H_2_O (OS2); samples submitted to 100 mM H_2_O_2_/10 µM FeSO_4_•7H_2_O (OS3); samples submitted to 1 M H_2_O_2_/100 µM FeSO_4_•7H_2_O (OS4); and samples submitted to 8 M H_2_O_2_/800 µM FeSO_4_•7H_2_O (OS5).

Figure 3 shows the DNA oxidation quantification using the 8-OHdG immunodetection assay and flow cytometry. Each histogram corresponds to the MIF of Alexa Fluor 488 obtained by incubating the sperm samples with different concentrations of the oxidising agent. It is evident that the fluorescence intensity increases with increasing concentration of the oxidising solution, being more evident when the sample was incubated with the highest oxidation concentration (8 M H_2_O_2_/800 µM FeSO_4_•7H_2_O), but these differences were not significant (*p* ≥ 0.05).

### 3.2. Sperm Chromatin Structure Assay (SCSA^®^)

**Figure 4:**
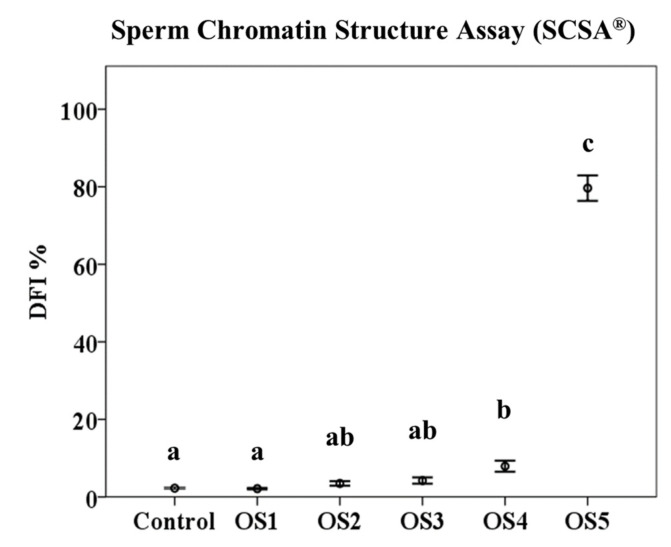
Effects of oxidant treatments on Sperm Chromatin Structure Assay (SCSA^®^) results. Mean of DNA fragmentation index (DFI %) ± standard error of the mean (mean ± SEM). a, b and c indicate significant differences (*p* < 0.05). OS1 = 1 mM H_2_O_2_/0.1 µM FeSO_4_•7H_2_O; OS2 = 10 mM H_2_O_2_/1 µM FeSO_4_•7H_2_O; OS3 = 100 mM H_2_O_2_/10 µM FeSO_4_•7H_2_O; OS4 = 1 M H_2_O_2_/100 µM FeSO_4_•7H_2_O; and OS5 = 8 M H_2_O_2_/800 µM FeSO_4_•7H_2_O.

In the analysis of SCSA^®^, we took into account two parameters: DFI % and HDS %. Our results showed that the percentage of DFI significantly increased (*p* < 0.05) between OS5 (79.66 ± 0.51%) and the rest of the treatments: control = 2.27 ± 0.03%; OS1 = 2.13 ± 0.03%; OS2 = 3.48 ± 0.09%; OS3 = 4.22 ± 0.13%; and OS4 = 7.92 ± 0.22%. Moreover, there were also differences (*p* < 0.05) between the OS4, control, and OS1 treatments (Figure 4).

**Figure 5:**
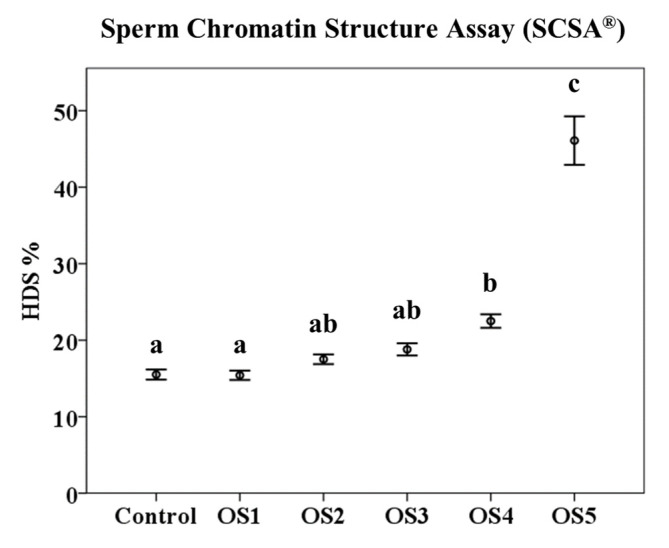
Effects of oxidant treatments on Sperm Chromatin Structure Assay (SCSA^®^) results. Mean of high DNA stainability (HDS %) ± standard error of the mean (mean ± SEM). a, b and c indicate significant differences (*p* < 0.05). OS1 = 1 mM H_2_O_2_/0.1 µM FeSO_4_•7H_2_O; OS2 = 10 mM H_2_O_2_/1 µM FeSO_4_•7H_2_O; OS3 = 100 mM H_2_O_2_/10 µM FeSO_4_•7H_2_O; OS4 = 1 M H_2_O_2_/100 µM FeSO_4_•7H_2_O; and OS5 = 8 M H_2_O_2_/800 µM FeSO_4_•7H_2_O.

The percentage of HDS followed the same pattern, and OS5 showed the highest values (*p* < 0.05; 46.09 ± 0.49) compared to the rest of the treatments (OS1 = 15.41 ± 0.09%; OS2 = 17.51 ± 0.10%; OS3 = 18.80 ± 0.12%; and OS4 = 22.50 ± 0.14%) and the control (15.51 ± 0.10%). There were also significant differences (*p* < 0.05) between the OS4, control, and OS1 treatments (Figure 5).

**Figure 6:**
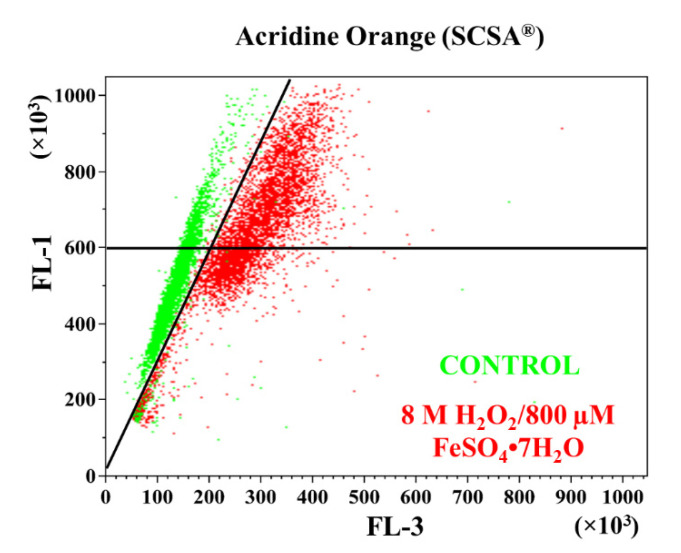
Assessment of sperm chromatin status. Cytogram obtained after carrying out the SCSA^®^ protocol. Green dots correspond to a sample that has not been treated with oxidant solution, and red dots correspond to a sample submitted to high exogenous oxidative stress. Dots plotted to the right of the diagonal line have increased DNA fragmentation index (DFI), and dots plotted above to the horizontal line have increased high DNA stainability (HDS).

Figure 6 shows the assessment of sperm chromatin status through SCSA^®^. The green dots correspond to samples that were not treated with oxidant solution (control), and the red dots correspond to samples submitted to high exogenous oxidative stress (8 M H_2_O_2_/800 µM FeSO_4_•7H_2_O). The dots plotted to the right of the diagonal line have increased DFI, and the dots plotted above the horizontal line have increased HDS. Consequently, the dots to the right of the diagonal line and the dots above the horizontal line correspond to the sample incubated with the highest oxidation concentration (OS5), demonstrating that the incubation of ram sperm with 8 M H_2_O_2_/800 µM FeSO_4_•7H_2_O increased the percentage of DFI and HDS in these samples.

## 4. Discussion

Oxidative stress is considered one of the most important factors regulating the vitality and functionality of mammalian spermatozoa [33]. These cells are highly vulnerable to oxidative stress due to their small cytoplasmic space, which leads to poor antioxidant protection and a great number of substrates that are vulnerable to a free radical attack, such as DNA and unsaturated fatty acids. Therefore, in this cell type, increased oxidative stress can lead to decreased motility, a loss of the ability to undergo the acrosome reaction, a decreased ability to fuse with the vitelline membrane of the oocyte, and also DNA damage [34].

The study of DNA damage in spermatozoa is particularly relevant as this damage is highly correlated with decreased fertilisation rates, a possible impairment of pre-implantation embryo development, an increased probability of early pregnancy loss, and a low fertility rate after conception [4,35,36].

In order to determine DNA damage in spermatozoa explicitly caused by oxidative stress, detection techniques were developed for 8-hydroxy-2′-deoxyguanosine (8-OHdG), which is an early marker of DNA oxidation [21]. To carry out the detection of 8-OHdG residues, different analytical methods using binding proteins [21] or antibodies [37] were developed. Vorilhon et al. (2018) evaluated human sperm samples with three different immunofluorescence methods to standardise a protocol for the specific immunodetection of 8-OHdG, using light microscopy, fluorescence microscopy, and flow cytometry and compared its efficacy with the commercial OxyDNA Test^®^ kit based on binding proteins [20]. In this study, the immunoassay-based protocols showed consistent reliability. Nevertheless, the microscopy-based protocols were much more time-consuming, and there was potential for variability in labelling sperm with 8-OHdG, in contrast to the flow cytometry-based protocol, which allowed a high number of cells to be analysed in a short time and was impartial. However, the protocol using the OxyDNA Test^®^ showed poor sensitivity and specificity when high levels of exogenous 8-OHdG were present [20]. Similar results were previously reported by Cambi et al. (2013), raising the question of the relevance of the OxyDNA assay’s conjugate for DNA 8-OHdG [38].

The SCSA^®^ technique is commonly used to assess ram sperm DNA damage. Because of Vorilhon et al.’s (2018) work, we wondered if we were underestimating DNA damage and not detecting possible oxidative stress-induced damage that the SCSA technique was not detecting [20]. For the reasons above, we proposed our study to evaluate DNA damage in ovine spermatozoa against increasing concentrations of oxidation using two techniques: SCSA^®^ and the detection of 8-OHdG content, following a specific immunoassay and flow cytometry protocol for ram sperm developed by Soria-Meneses et al. (2019) [25]. As an oxidising agent, we used H_2_O_2_ because, in combination with FeSO_4_•7H_2_O, it acts as a reducing agent and generates hydroxyl radicals (OH•) [39], inducing the production of DNA damage [40].

On the one hand, the 8-OHdG immunodetection results did not show significant differences between the MIF resulting from the incubation with different concentrations of the oxidising agent. Although these differences were not significant, probably due to the high value of the standard error of the mean, there is evidence that the highest concentration of the oxidant solution (8 M H_2_O_2_/800 µM FeSO_4_•7H_2_O) could affect DNA oxidation. These results contrast with data obtained in other studies on detecting 8-OHdG adducts in sperm DNA, in which lower concentrations of the oxidising agent were used as positive controls [4,20,21,38,41,42,43]. De Iuliis et al. (2009) exposed human spermatozoa to increasing concentrations of H_2_O_2_ and Fe^2+^, resulting in a linear response to the formation of 8-OHdG [21]. A linear response in the 8-OHdG formation was also found in human sperm after incubation with 25 µM H_2_O_2_ [38], 4 M H_2_O_2_ [41], and 8 M H_2_O_2_ [20]. In addition, Serafini et al. (2018) incubated stallion sperm with different DNA damage-enhancing media, including an oxidising agent (10 µM FeSO_4_/20 µM H_2_O_2_), and obtained a higher percentage of sperm with 8-OHdG than control samples [42]. Zhu et al. (2017) showed a lower 8-OHdG content in rabbit sperm under the oxidising conditions of 2 mM H_2_O_2_/1 mM FeCl_2_•4H_2_O in response to the antioxidant cysteine [43]. However, our results show that, in ram sperm samples, only the highest concentration of oxidising agents (8 M H_2_O_2_/800 µM FeSO_4_•7H_2_O) achieve higher MIF values in the detection of DNA damage by the 8-OHdG immunodetection assay.

On the other hand, the SCSA^®^ results showed that both the DFI % and HDS % were significantly different between the samples that were incubated with high concentrations of the oxidant solution (1 M H_2_O_2_/100 µM FeSO_4_•7H_2_O and 8 M H_2_O_2_/800 µM FeSO_4_•7H_2_O) and the rest of concentrations. Furthermore, lower concentrations of the oxidising agent do not seem to affect DNA integrity in ram sperm samples [44]. Due to the high degree of DNA compaction in ram sperm, physiological concentrations of free radicals do not affect its integrity, as shown in a recent work by Peris et al. (2019), where incubating spermatozoa in capacitating conditions did not affect DNA integrity [45]. No significant differences in DNA integrity have been found in other ruminant species such as goat [46] and red deer [47,48,49,50]. These results are in the contrast with human spermatozoa, where a change in environment, exposure to polycyclic aromatic hydrocarbons (PAHs), and certain pathologies could increase DFI % and HDS % [10,17,19,51].

The results in the present study revealed that, under high oxidative stress conditions, the integrity in ovine sperm DNA is impaired, and both the 8-OHdG immunodetection assay and SCSA^®^ can detect this damage. Unless a pathological process is involved, DNA damage is rarely found in small ruminant sperm [52]. This might be because ram sperm only have P1-protamine, whereas horse, mouse, and human have both P1- and P2-protamines [53]. In addition, sperm chromatin from species expressing both protamines is more susceptible to decondensation; this property could also make ram sperm DNA more resistant to damage associated with oxidative stress [54].

It is assumed that, along with lipids in associated acrosomal membranes and the cytoplasmatic droplet, lipids in the plasma membrane are the main targets for attack by ROS. Mammalian spermatozoa are especially susceptible to ROS damage because of their high polyunsaturated fatty acid content. In ram spermatozoa, arachidonic, docosapentaenoic, and docosahexaenoic acids account for approximately 65% of the total phospholipid-bound fatty acids [55]. This may result in an increased production of 4-HNE, one of the products generated by lipid peroxidation, which has mitochondria as its main target, stimulating mitochondrial superoxide production. The activation of mitochondrial electron leakage by 4-HNE is involved in the disruption of succinate dehydrogenase activity, the subsequent activation of intrinsic apoptotic cascades, the loss of mitochondrial membrane potential and, eventually, the formation of oxidative DNA adducts, DNA strand breakage, and cell death [56].

In this study, we compared two techniques for detecting sperm DNA damage: an 8-OHdG immunodetection assay and SCSA^®^. These two techniques differ in their protocol and the parameters evaluated. While the 8-OHdG immunodetection assay allows us to determine the presence of 8-OHdG adducts in DNA, SCSA^®^ evaluates the DFI % and HDS %. SCSA^®^ is a low-complexity technique, in which samples are denatured before staining with the metachromatic fluorochrome AO and evaluated by flow cytometry. In contrast, the immunodetection of 8-OHdG requires several steps such as washing, treatment with lysis and blocking PBS, and incubation with antibodies, a procedure that could take several hours but can be detected with flow cytometry and fluorescence microscopy. Another difference is that 8-OHdG detection has a variable protocol, whereas SCSA^®^ has a protocol that does not vary depending on the species to be analysed [5,20].

Our study offers similar results using both assays; we observed an increase in the detection of 8-OHdG adducts and an increase in DFI % and HDS % only when the sperm samples were subjected to high concentrations of an oxidant solution or oxidative stress conditions that are not physiological. These results show that, for oxidative-stress-related sperm DNA damage in ovine sperm, both techniques are valid, probably due to the high DNA compaction in these samples. Regardless, this technique must be validated for each species and for each type of sperm sample.

## 5. Conclusions

An increase in oxidative stress conditions can lead to the oxidation of bases in DNA, thereby leading to the formation of 8-OHdG [57] but could also lead to DNA destabilisation and an increased susceptibility of DNA to hydrolysis, resulting in the formation of single-stranded DNA, which SCSA^®^ can assess. For these reasons, an oxidative-stress-specific assay such as 8-OHdG immunodetection is not needed to measure DNA damage caused by oxidative stress in ram sperm samples because a more straightforward technique such as SCSA^®^ also detects this damage and allows for the evaluation of a larger number of cells in a short period of time.

## Data Availability

The data underlaying this article will be shared on reasonable request to the corresponding author.
